# Mitochondrial Genome Sequence of the Legume *Vicia faba*

**DOI:** 10.3389/fpls.2013.00128

**Published:** 2013-05-07

**Authors:** Valentine Negruk

**Affiliations:** ^1^Biotechnology Research Lab, Miami Dade CollegeMiami, FL, USA

**Keywords:** *Vicia faba*, mitochondrial genome, sequencing, *Medicago truncatula*, nuclear genome

## Abstract

The number of plant mitochondrial genomes sequenced exceeds two dozen. However, for a detailed comparative study of different phylogenetic branches more plant mitochondrial genomes should be sequenced. This article presents sequencing data and comparative analysis of mitochondrial DNA (mtDNA) of the legume *Vicia faba*. The size of the *V. faba* circular mitochondrial master chromosome of cultivar Broad Windsor was estimated as 588,000 bp with a genome complexity of 387,745 bp and 52 conservative mitochondrial genes; 32 of them encoding proteins, 3 rRNA, and 17 tRNA genes. Six tRNA genes were highly homologous to chloroplast genome sequences. In addition to the 52 conservative genes, 114 unique open reading frames (ORFs) were found, 36 without significant homology to any known proteins and 29 with homology to the *Medicago truncatula* nuclear genome and to other plant mitochondrial ORFs, 49 ORFs were not homologous to *M. truncatula* but possessed sequences with significant homology to other plant mitochondrial or nuclear ORFs. In general, the unique ORFs revealed very low homology to known closely related legumes, but several sequence homologies were found between *V. faba, Beta vulgaris*, *Nicotiana tabacum*, *Vitis vinifera*, and even the monocots *Oryza* *sativa* and *Zea mays*. Most likely these ORFs arose independently during angiosperm evolution (Kubo and Mikami, 2007; Kubo and Newton, 2008). Computational analysis revealed in total about 45% of *V. faba* mtDNA sequence being homologous to the *Medicago truncatula* nuclear genome (more than to any sequenced plant mitochondrial genome), and 35% of this homology ranging from a few dozen to 12,806 bp are located on chromosome 1. Apparently, mitochondrial rrn5, rrn18, rps10, ATP synthase subunit alpha, cox2, and tRNA sequences are part of transcribed nuclear mosaic ORFs.

## Introduction

The number of plant mitochondrial genomes being sequenced exceeds two dozen. Sequencing data analysis was well presented in different reviews (Kubo and Mikami, 2007; Kubo and Newton, 2008; Kitazaki and Kubo, 2010; Maréchal and Brisson, 2010; Woloszynska, 2010). The published data indicate that flowering plants contain the largest mitochondrial genomes reported so far, consisting of heterogeneous populations of mainly linear and sometimes circular DNA molecules. Interestingly, size and organization of these molecules differ not only among closely related plant species but even among lines of the same species (Bendich, 1993; Oldenburg and Bendich, 1996; Kubo and Mikami, 2007; Kubo and Newton, 2008). Sequences of plant mitochondrial genomes in most cases are organized as large circular molecules called “master chromosome,” usually containing several large (>500 bp) direct and a few inverted repeats. It has been suggested that mitochondrial DNA (mtDNA) is replicated in a recombination-dependent manner (Oldenburg and Bendich, 1996; Backert and Börner, 2000; Kubo and Newton, 2008). Intra- and inter-molecular recombination between large repeats can cause different isomeric forms or subgenomic versions of a master chromosome (Kubo and Mikami, 2007; Kubo and Newton, 2008; Kitazaki and Kubo, 2010; Maréchal and Brisson, 2010; Woloszynska, 2010; Chang et al., 2013). However, mitochondrial heteroplasmy is mainly determined by homologous recombinations between short repeats (<500 bp) (Vitart et al., 1992; Hartmann et al., 1994; Kanazawa et al., 1994; Bellaoui et al., 1998; Janska et al., 1998; Arrieta-Montiel et al., 2001; Albert et al., 2003; Woloszynska and Trojanowski, 2009; Alverson et al., 2011).

Differences in master chromosome size are due to unique sequences present in one and absent in another species’ mtDNA, or to repeat sequences or large duplications (mostly >1000 bp) representing up to 35% of the total genome size. For example, mitochondrial genome sizes of *Zea mays* lines range from 535,825 to 739,046 bp, while genome complexities range from 506,760 to 537,180 bp (Allen et al., 2007). Even unique sequences of mitochondrial genomes may differ intraspecifically by up to 7%.

Recombination between short repeats can lead to gain or loss of sequences. Sequence gain might come from chloroplast or nuclear genomes of the same plant, sometimes from mitochondrial plasmid DNA (Kubo et al., 2000; Kubo and Newton, 2008; McDermott et al., 2008; Kitazaki and Kubo, 2010) or even from viruses (Marienfeld et al., 1997; Goremykin et al., 2009), fungi, bacteria, or other plants. Sequence loss from mitochondrial genomes can be compensated by transfer to the nuclear genome (Kubo and Newton, 2008; Kitazaki and Kubo, 2010; Alverson et al., 2011).

Plant mitochondrial heteroplasmy covers a significant part of the mitochondrial genome. Less than 20% of the genome is represented by known protein, rRNA and tRNA encoding genes. The coding parts of these sequences are highly conserved. Lists of tRNA were found different for various plants, but every tRNA sequence is conserved and might have a mitochondrial or chloroplast origin (Marienfeld et al., 1997; Kubo and Newton, 2008; Kitazaki and Kubo, 2010; Alverson et al., 2011). In addition to known genes, every plant mitochondrial genome has an additional 10% or more of putative open reading frames (ORF). Some of these frames are conserved across several plant species, while others can be unique. Recombination events between short repeats in there majority do not alter the known coding sequences and ORFs. However, cases of altered ORFs or coding sequences were found (Marienfeld et al., 1997; Kubo and Newton, 2008; Kitazaki and Kubo, 2010; Alverson et al., 2011), some of these cause cytoplasmic male sterility (CMS). The mechanisms of CMS differ and are specific for each case (Allen et al., 2007; Kubo and Newton, 2008; Kitazaki and Kubo, 2010).

The study of plant mitochondrial genomes revealed important information regarding the evolution of these genomes (Kitazaki and Kubo, 2010) and of entire eukaryotic systems as well. Every plant mtDNA has some sequences in common with that of all other plants, as well as species- or group-specific sequences. Closely related plants usually share significant portions of mitochondrial sequences, but in some cases their mitochondrial sequences differ remarkably. More sequencing data are needed to supply sufficient information for a detailed comparative study of different phylogenetic groups of plants. Here sequencing data and comparative analysis are provided for the mitochondrial genome of the legume *V. faba*.

## Materials and Methods

### Mitochondrial DNA isolation, library construction, genome sequencing, and assembly

Mitochondria were isolated from 6 to 7 days, dark grown, etiolated seedlings of *V. faba* cultivar Broad Windsor (Territorial Seed Company, Cottage Grove, OR, USA) using DNAse I protocol. Purified mitochondria were lyzed and mtDNA was isolated as reported (Synenki et al., 1978). Three libraries were constructed:

1. mtDNA was digested by *Bam*HI and cloned in *Bam*HI digested pUC19 plasmid vector.
2. mtDNA was digested by *Apo*I and cloned in *Eco*RI digested pUC19 plasmid vector.
3. A third library was generated by Genomex Biotechnology Company (Genomex appears to be a trading name of Amplicon Express, http://www.amplicon-express.com); mtDNA was mechanically nicked and 25–45 kb DNA fragments were cloned into the fosmid vector pEpiFOS-5. This library contains ∼1500 clones with an average insert size of 35 kb.

About 400 *Bam*HI fragments were sequenced from both strands with sequence overlap of at least 100 bp for each primer. “Difficult” sequences with compression or large number of homonucleotide stretches were sequenced a few more times using different primers for both strands until the sequence became clear. For sequencing an Applied Biosystems four-capillary sequencing machine 3130 Genetic Analyzer with 55 cm column and corresponding sequencing kits was used. Among 400 sequenced fragments were 119 unique *Bam*HI fragments.

From the *Apo*I library we isolated 1050 clones; 158 of these were selected containing at least one *Bam*HI recognition site (∼30% of them contained inserts with two or three *Bam*HI sites). These clones were thoroughly sequenced. Most of them overlapped partially or fully with already sequenced *Bam*HI fragments and revealed the arrangement of these fragments. *Apo*I library sequencing led to additional 11 *Bam*HI fragments.

Finally, 234 long fosmid clones ranging from 25 to 45 kb with average around 35 kb were sequenced. For direct sequencing of these fragments at a good quality, the PCR program was: 95°C for 5 min, followed by 50 cycles of 95°C for 30 s, 55°C for 10 s, 60°C for 4 min, and hold at 4°C.

For most of the sequencing cycles, primers known from *Bam*HI and *Apo*I library sequencing were used, but at times new primers corresponding to new *Bam*HI fragments were applied. By sequencing these fosmid DNA fragments 33 new unique *Bam*HI fragments mainly representing recombinant versions of known fragments were found.

In total∼9 × 10^6^ bp of *V. faba* mtDNA were sequenced corresponding a 15-fold coverage of the master chromosome. Of 163 unique *Bam*HI fragments 144 could be included into the master chromosome sequence. Nineteen other fragments were recombinant versions between some of the 144 *Bam*HI fragments belonging to the master chromosome. Computer alignment of all *Bam*HI fragments and 234 fosmid DNA inserts allowed the construction of a 588,000 kb circular master chromosome that contained all unique sequences of *V. faba* mtDNA and was submitted to the GenBank database with accession number KC189947. Computation analysis was conducted using NCBI tools.

## Results and Discussion

### *Vicia faba* mitochondrial DNA sequencing data compared to sequences earlier reported

Comparative analysis of our data and results previously published by Wahleithner and Wolstenholme (1988b), MacFarlane et al. (1990a,b), and Wahleithner et al. (1990), as expected, showed very high similarity. Few single nucleotide substitutions were found mainly in non-coding regions. Protein sequences of *cob* (392aa), *atp9* (both 88 and 74aa), *cox3* (265aa), and *nad1* (325aa) were 100% identical. The *atp6* protein (291aa) sequences had just one amino acid difference – isoleucine versus leucine.

A difference was found between rps14 coding sequences (100 amino acid length). In our version, it was glycine in position 85 instead of serine reported by Wahleithner and Wolstenholme (1988b). Multiple alignments between ribosomal protein S14 sequence and protein database showed that glycine is a standard amino acid on this position. The *V. faba* sequence is identical to *rps14* of *Pisum sativum* (Hoffmann et al., 1999). The minor differences between known *rps14* of different plant mitochondria never concerned glycine in this position.

Significant differences of mtDNA sequences between cultivar Broad Windsor and another cultivar of *V. faba* were found as reported (Scheepers et al., 1997) around ORF143 near exon c of the *nad5* gene. Actually, the Broad Windsor mitochondrial genome lacks a full size ORF143. Instead, it has ORF295 and ORF245. Amino acid sequences for nad5 protein exons c, d, and e related are identical.

### The master chromosome structure

**Figure 1:**
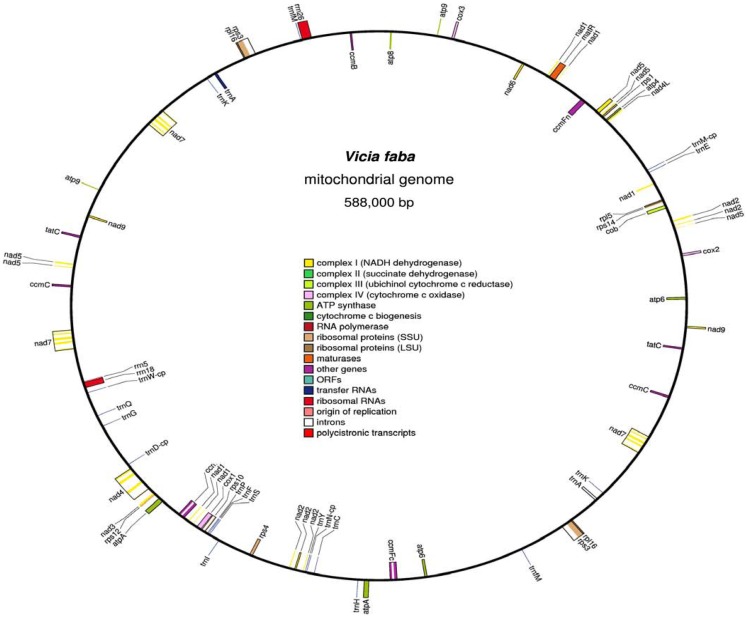
**The circular map of *Vicia faba* mitochondrial genome**. Clockwise and counter-clockwise strands are drawn on the inside and outside of the outer circle, respectively. GC content is shown in the inner circle. The map was drawn by using OGDRAW (Bock et al., 2007).

Computer alignment of all *V. faba* mitochondrial *Bam*HI fragments and 234 mtDNA fragments (25–45 kb) cloned in fosmids allowed to construct a 588,000 kb circular master chromosome with 45.04% GC content (Figure 1)*. V. faba* shared about 40% similarity with mtDNA of the legumes *Lotus japonicus*, *Millettia pinnata* (Kazakoff et al., 2012), *Glycine max* (Chang et al., 2013), and *Vigna radiata* (Alverson et al., 2011). For all other known plant mitochondrial genomes homology was 25% and lower. Eleven large (>500 bp) repeats were found in the master chromosome: eight direct and three inverted ones. The largest repeat comprises 66,893/66,897 bp, the smallest 1,675 bp. Large repeats were highly similar (99%) or identical. Ten repeats have two, and one (the smallest) has three copies. The total size of large repeats covers 200,255 bp or 34% of the whole master chromosome size. The contribution of short (<500 bp) repeats has not been calculated but should not change significantly the complexity of the *V. faba* mitochondrial genome of 387,745 bp.

Three types of protein or putative polypeptide related sequences were found in *Vicia faba* mtDNA:

1. Conservative sequences, common to all other plants.
2. Aberrant putative ORFs unique to the specific plant or to a small group of plants.
3. Short fragments of known polypeptides which are not organized into ORF.

Thirty five mitochondrial genes encoding conserved proteins, 3 ribosomal RNAs and 17 tRNA genes were found. The *V. faba* master chromosome contained all nine *nad* genes of Complex I, the *cob* gene of Complex III, three *cox* genes of Complex IV, five *atp* genes of Complex V, four *ccm* genes of cytochrome c biogenesis, and 7 out of 16 possible ribosomal protein genes. For *sdh3* and *sdh4* genes of Complex II and for *rps7* only fragments encoding the corresponding polypeptide sequences were found. For *sdh3* and *sdh4* these fragments appeared as parts of unknown ORFs.

Amino acid sequence alignment revealed an 110aa rps7-related mitochondrial polypeptide for *Pisum sativum* and *Lotus japonicus*. In *V. faba* it was divided by a stop codon instead of serine in position 73 into two polypeptides of 72 and 37aa which were not organized into ORF. The genes *nad9*, *atp6*, *atp9*, *ccmC*, *rpl16*, *rps3*, and *tatC* were duplicated. Gene *nad5* exons D and E were also duplicated. The gene *nad7* with all five exons was triplicated. Additionally, exon 5 of *nad7* was copied to other locations.

Comparative analysis between the master chromosome and minicircles (Wahleithner and Wolstenholme, 1987) did not reveal any significant sequence homology longer than a few dozen nucleotides. The origin and direction of replication for *V. faba* mitochondrial plasmids determined by Wahleithner and Wolstenholme (1988a) were aligned with the master chromosome sequence. Few dozens of 10–13 bp sequences, homologous to the core motif AGGAA, with few nucleotides flanking this motif were found on the master chromosome.

Six of the tRNA genes (Table 1) were highly homologous to chloroplast genome sequences.

**Table 1:** **tRNA genes found in the mtDNA of *Vicia faba***.

Amino acid	Codon	tRNA	Anticodon
Trp	UGG	cp-trnW**	CCA
His	CAC	cp-trnH	GUG
Ala	GCU	cp-trnA x2	UGC
Ile*	AUG	cp-trnM	CAU
Gln	CAA	mt-trnQ	UUG
Tyr	UAC	mt-trnY	GUA
Lys	AAA	mt-trnK x2	UUU
Gly	GGC	mt-trnG	GCC
Asn	AAC	cp-trnN	GUU
Asp	GAC	cp-trnD	GUC
Cys	UGC	mt-trnC	GCA
Met	AUG	mt-trnM	CAU
fMet	AUG	mt-trnM x2	CAU
Pro	CCA	mt-trnP	UGG
Phe	UUC	mt-trnF	GAA
Ser	AGC	mt-trnS	GCU
Glu	GAA	mt-trnE	UUC

*Suggesting that methionine anticodon is post-transcriptionally modified to provide tRNA with isoleucine activity (Michaud et al., 2011).

**cp-tRNA on this table means that we found similar sequences in other plant chloroplast genome. Some of them are not 100% identical to corresponding chloroplast tRNA (see Section Results).

### Retrotransposon-related sequences of *V. faba* mitochondrial DNA

All three classes of nuclear retrotransposon-related sequences were found within the *V. faba* mitochondrial genome; the Ty1/*copia* subclass, a non-LTR retroelement reverse transcriptase and an ORF with notable homology to the RNase H and reverse transcriptase domains of the Ty3/*gypsy* superfamily. These sequences were represented by relatively short ORFs (encoding less than 200aa) with high similarity to large nuclear ORFs (>1000aa), similar as described previously (Knoop et al., 1996).

### Mitovirus related sequences

The open reading frame ORF128, in position 4678–5064 of *V. faba* mtDNA revealed high similarity to the RNA-dependent RNA polymerase region of virus pfam05919 belonging to Mitoviruses of the family *Narnaviridae*. Mitoviruses are simple viruses that invade fungal mitochondria without forming true capsids (Cole et al., 2000). Their genomes consist of one gene encoding RNA-dependent RNA polymerase. ORFs representing part of mitoviral RNA polymerase were found in mitochondrial genomes of *Arabidopsis thaliana* (Marienfeld et al., 1997; Hong et al., 1998), *Brassica napus* (Tuomivirta and Hantula, 2005), and *Vitis vinifera* (Goremykin et al., 2009). A small part of mitovirus related sequences was reported previously for *V. faba* mtDNA (Marienfeld et al., 1997).

### Open reading frames of *V. faba* mitochondrial DNA

The total number of genes encoding conservative proteins, rRNAs, and tRNAs was 52. In addition, we found 114 unnamed ORF, in there majority more than 100aa long. NCBI BLAST analysis revealed three groups of ORFs:

1. 36 ORFs with no significant homology to any known proteins
2. 29 ORFs with significant homology to *Medicago truncatula* nuclear genome. Some of them were also homologous to other plant mitochondrial ORFs
3. 49 ORFs with significant homology to other plant mitochondrial or nuclear ORFs.

Some ORFs from groups 2 and 3 might represent novel alternative splicing variants formed by exonization of non-coding DNA sequences (Chen et al., 2012). Other ORFs consisted in part of known conservative protein encoded by non-mosaic genes together with parts of unknown sequences as part of ORFs from *V. faba*, or from other plant mitochondria.

### *V. faba* ORFs with significant homology to other plant mitochondrial ORFs

NCBI BLAST analysis of unique mtORFs of *V. faba* revealed very low similarity to those of known closely related legumes. Three different *V. faba* ORFs showed homology to parts of *Millettia pinnata* sdh3, one to the ORF90 of *Lotus japonicus* and none to *Vigna radiata*. Nevertheless, a several sequence homologies were found between *V. faba*, *Beta vulgaris*, *Nicotiana tabacum*, *Vitis vinifera*, and even the monocots *Oryza* *sativa*, *Zea mays* (Table 2).

**Table 2:** **ORF comparison between *Vicia faba* and some other plant mitochondrial genomes**.

*Vicia faba*	*Vitis vinifera*	*Beta vulgaris*	*Nicotiana tabacum*	*Arabidopsis thaliana*	*Oryza sativa*	*Zea mays*	*Lotus japonicus*	*Millettia pinnata*	*Vigna radiata*	*Vicia faba*
ORF295	-	ORF187	ORF171	ORF118	ORF288	ORF179	-	sdh3	-	ORF143*
		ORF324		ORF307						
		ORF297		ORF216						
		ORF256		ORF297						
		ORF99								
		ORF237								
ORF145	-	ORF134c	-	-	-	-	-	-	-	
ORF245	-	ORF169	-	ORF313	-	-	-	-	-	
				ORF215							
ORF101	-	-	-	ORF145	-	-	-	-	-	
ORF102	ORF104	-	ORF103	-	-	ORF105	-	-	-	
ORF107	-	ORF124	ORF125d	-	-	-	-	-	-	
ORF68	sdh4	-	ORF125e	-	-	-	-	-	-	
ORF221	-	-	-	-	-	-	-	-	-	ORF128**
ORF90	-	-	-	-	-	-	ORF90	-	-
ORF245	-	ORF187	ORF171	ORF118	ORF288	ORF179	-	-	-	ORF143*
		ORF324		ORF307							
		ORF297		ORF216							
		ORF256		ORF297							
		ORF99								
		ORF237								
ORF103	-	-	-	-	-	-	-	sdh3	-	ORF128**
ORF126	-	-	ORF177	-	-	-	-	-	-	
ORF184	ORF185	+	ORF125f	-	-	-	-	-	-	
ORF101	-	-	-	ORF145b	-	-	-	-	-	
ORF177	-	ORF310	-	-	-	-	-	-	-	
		ORF270									
		ORF170									
ORF110	-	ORF124	ORF125d	-	-	-	-	-	-	
ORF167	psbA	ORF227	ORF274	-	-	-	-	-	-	
		ORF224	ORF315								
		ORF198								
ORF109	RNApol	ORF598	-	-	-	ORF417	-	-	-	
		ORF1014								
ORF301	psbA	ORF227	ORF274	-	-	-	-	sdh3	-	
			ORF315								
ORF142	-	-	ORF101b	-	-	-	-	-	-	
ORF115	-	ORF125b	-	-	-	-	-	-	-	
ORF321	-	-	-	ORF161	-	-	-	-	-	

ORF143* was reported by Scheepers et al. (1997). It was not found in mtDNA of *Vicia faba* cultivar Broad Windsor.

ORF128** was reported by MacFarlane et al. (1990b). It is incomplete ORF. We found that in one version it was ORF129, a part of sub genomic linkage group. On the other hand, ORF129 is a part of ORF221 which was found in master chromosome.

*V. faba* ORF143 was reported by Scheepers et al. (1997). Both Ad/N and Ad/447 line mitochondria possessed ORF143 but it was not found in Broad Windsor. Instead we found two longer ORF295 and ORF245. In the case of ORF295, the first 28 amino acids are highly homologous to the first 28 amino acids of nad3. The central part of ORF245 has a high homology, with the central part of ORF295 but both N and C ends of these ORFs are different. Note that both *Beta vulgaris* and *Oryza sativa* mitochondria also possess multiple ORFs, partially homologous to *V. faba* ORF295, ORF245, and ORF143 (Table 2). We also found that some of our unique ORFs were homologous to ORFs in mitochondrial genomes of *Daucus carota, Citrullus lanatus, Lupinus luteus, Brassica napus, Boea hygrometrica, Phoenix dactylifera, Glycine max, Phaseolus vulgaris*.

### Chloroplast-specific insertions in *Vicia faba* mitochondrial DNA

The chloroplast genome of *V. faba* has not yet been sequenced. Therefore, chloroplast sequences of *Medicago truncatula, Arabidopsis thaliana*, *and Glycine max* were used to find chloroplast-derived insertions in *V. faba* mtDNA. Analysis of *M. truncatula* chloroplast-specific (cp) sequences revealed 10 fragments (four of them duplicated) in *V. faba* mtDNA, ranging from 77 to 1389 nt with similarity of 74–97%. The sequences homologous to cpDNA comprise 1.1% of *V. faba* mtDNA. Six of the 10 cpDNA fragments contained tRNA genes (*tRNA^Ala^*, *tRNA^Trp^*, *tRNA^Asn^*, *tRNA^Asp^*, *tRNA^His^*, and *tRNA^Met^*). The chloroplast-encoded *tRNA^Ala^* gene contained one intron. Both exons of *tRNA^Ala^*, as well as the *tRNA^Trp^* gene sequence, were 100% homologous to *V. faba* mtDNA. For four other cp-tRNA genes the identity was <100%. Almost all of these sequences were also found within the *M. truncatula* nuclear DNA. In addition to the tRNA genes, cp, and mtDNA share homologous sequences encoding fragments of 16S and 23S ribosomal RNA as well as fragments of proteins rpl12 and ycf68.

### Sequence homology between *Vicia faba* mitochondrial DNA and *Medicago truncatula* nuclear genome

Homology analysis between *V. faba* mtDNA and the nuclear genome of *A. thaliana* using NCBI BLAST search revealed about 20% of homologous mtDNA. For the related legume *Glycine max*, the homology was slightly higher (∼27%).

A high level of co-linearity was found earlier between the nuclear linkage groups of the legumes *V. faba* and *M. truncatula*, despite the large differences in genome size (Ellwood et al., 2008; Young et al., 2011; Alghamdi et al., 2012). Chromosome mapping demonstrated an evidence of shared macrosynteny between *V. faba* and *M. truncatula* nuclear genomes (Ellwood et al., 2008). The nuclear genome of *M. truncatula* has recently been sequenced (Young et al., 2011). So, it was logical to look at possible similarities between *V. faba* and *M. truncatula* on the nucleotide and amino acid sequence level. NCBI BLAST search revealed about 45% of *V. faba* mtDNA sequence being homologous to *M. truncatula* nuclear sequences. This is more homology than found with any sequenced plant mitochondrial genome. Thirty five percent of homologous sequences range from a few dozen to 12,806 bp and are located on chromosome 1.

**Figure 2:**
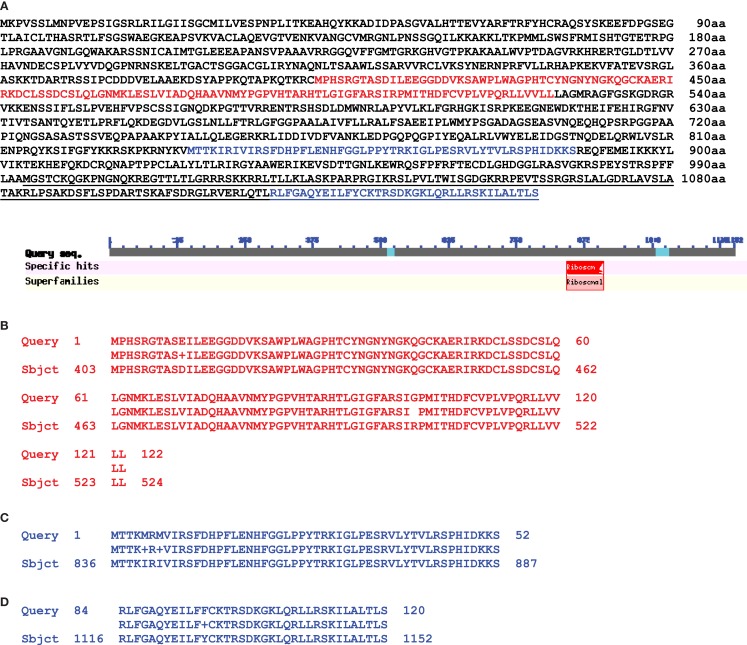
**Protein alignment between *M. truncatula* nuclear putative ORF1152 and *Vicia faba* mitochondrial ORF135 and gene rps10**. **(A)** Amino acid sequence of *M. truncatula* chromosome 1 putative ORF1152 (NCBI BLAST). **(B)** Red colored sequence which is a part of mitochondrial ORF135. **(C,D)** Blue colored parts of the sequence which is a *Vicia faba* CDS of rps10 gene. **(C)** An exon 1 sequence and **(D)** is an exon 2 sequence. Underlined is a sequence of nuclear alternative gene encoding putative rps10 (159aa).

**Figure 3:**
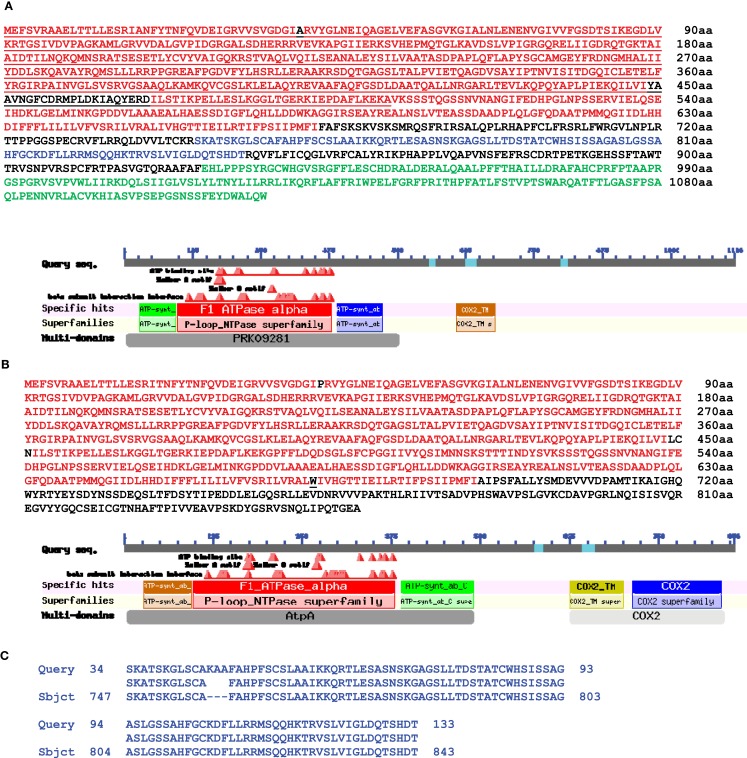

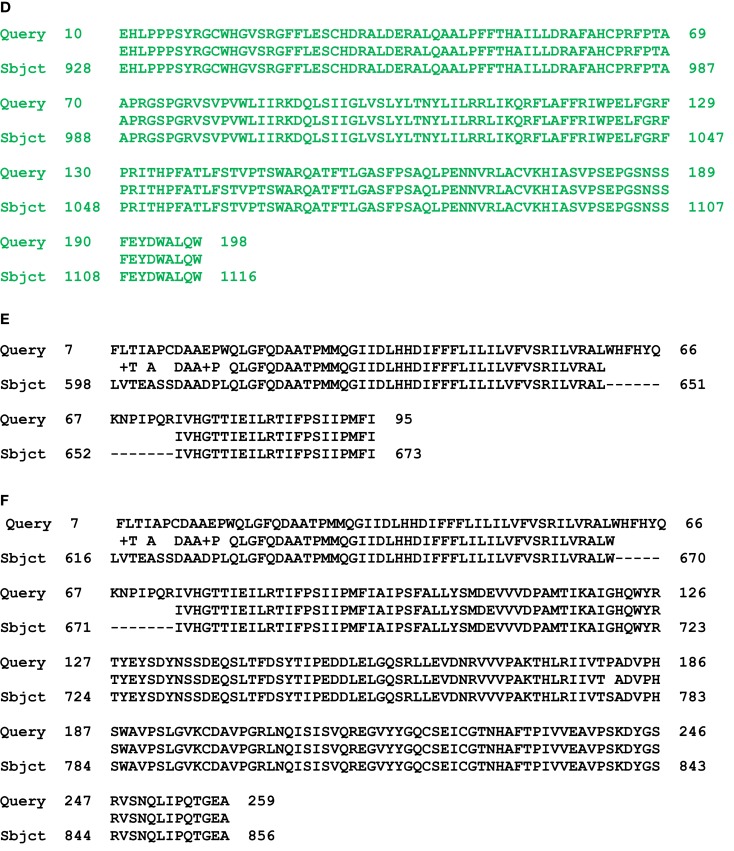
**Protein alignment between *M. truncatula* nuclear putative ORF1116 and ORF856 and *Vicia faba* mitochondrial genes atpA and cox2**. **(A)** Amino acid sequence of *M. truncatula* chromosome 1 putative ORF1116 and **(B)** ORF856 (NCBI BLAST). Red colored is a homologous sequence between ORF1116 and ORF856. Black colored are different amino acids within area of homology between ORF1116 and ORF856 (red colored). Underlined is a perfect (99%) homology between *Vicia faba* mitochondrion gene of ATP synthase F1 subunit 1 and ORF1116. **(C)** A blue colored homology between mitochondrial ORF134 and ORF1116. **(D)** A green colored homology between mitochondrial ORF198 and ORF1116. **(E)** Homology between *Vicia faba* mitochondrion cox2 gene (259aa) and ORF1116. **(F)** Homology between *Vicia faba* mitochondrion cox2 gene (259aa) and ORF856.

In this publication we present some data interesting in aspect of sequence relationships between mitochondrial and nuclear genomes. When we analyzed homology between these two genomes in the area of mitochondrial 5S (*rrn5*), 18S (*rrn18*), and *rps10* genes we found significant (99%) DNA sequence homology overlapping 5S, 18S, ORF134, ORF198, trnW-cp, and about 8000 bp of following uninterrupted sequence homology (positions, complement 320126–333187 bp). We found in this area large ORF1152 annotated as putative ribosomal protein S10 in *Medicago truncatula* chromosome 1 (Figure 2) (sequence encoding this ORF overlapped genes *of rrn5* and *rrn18* ribosomal RNA with 99% homology as well as ORF135). It was a transcribed mosaic gene with 18 exons. ORF1152 amino acid sequence was fused with *V. faba* mtDNA gene of *rps10* highly homologous to mitochondrial genomes of many plants (position, complement (383419–384753). Thus, we found a transcribed nuclear genome sequence organized into ORF1152, which contained sequences of *rrn5* and *rrn18*, and fused with amino acid sequence covering mitochondrial *rps10* gene located 63000 bp apart. And it was not just a single case. We found two more transcribed ORFs: ORF1116 and ORF856, in different positions of *Medicago truncatula* chromosome 1 (Figure 3). First halves of these ORF are similar and alternatively spliced. Second halves are different. Both of them are fusions between ATP synthase subunit alpha and *cox2* genes but for ORF856 homology to *cox2* gene was much more significant (Figure 3). In addition to ATP synthase subunit alpha and *cox2* genes two unnamed protein products ORF134 and ORF198 were found in *V. faba* mtDNA. ORF134 and ORF198 were homologous to second half of ORF1116 but not to ORF856. At the same time, ORF1152 and ORF1116 nucleotide sequences shared around (99%) of 1700 bp complementary nucleotide sequence homology.

### tRNA related sequences in *Medicago truncatula* nuclear genome

**Figure 4:**
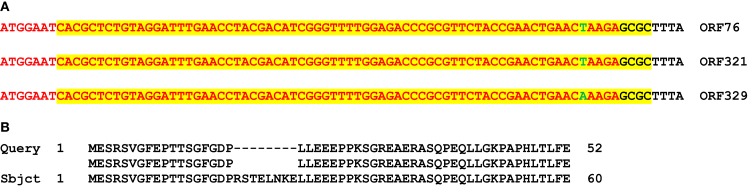
**Nucleotide (A) and protein (B) alignment between *M. truncatula* nuclear putative ORF76, ORF321, and ORF329 and *Vicia faba* mitochondrial gene tRNA^Trp^**. **(A)** A nucleotide sequence of exon 1 (red color) in putative ORF76, ORF321, and ORF329 (NCBI BLAST). Highlighted with yellow is tRNA^Trp^ in complementary orientation. Black color is intron sequence. Green color shows point mutation. **(B)** An alignment between exon 1 (first 18 amino acids) and part of exon 2 sequences. Query is a sequence of ORF76 or ORF321, Sbjct is a sequence of ORF329.

Here we present data only for tRNAs found common for chloroplast and mitochondrial genomes. For mitochondrial *tRNA^Ala^*, we found homology with *M. truncatula* nuclear genome only for exon 2 of *tRNA^Ala^*. All other full size tRNA sequences common both for chloroplast and mitochondrial genomes were found in *M. truncatula* nuclear genome. Four copies of *V. faba* tRNA^Trp^ sequence found in chromosome 1, and 1 copy in chromosome 3, 4, and 8 each. In chromosome 1, *tRNA^Trp^* sequence was found as a part of genes encoding ORF76 (2 copies in opposite orientation), ORF321 and ORF329. All three genes were transcribed and had mosaic structure. ORF76 had 2 exons, ORF321 and ORF329 had 6 exons each. For ORF76 and ORF321 positions of exon 1, following intron and exon 2 were the same. In all three ORFs sequence complementary to *tRNA^Trp^* gene covered exon 1, starting from nucleotide 8 until the end, and part of the following intron. For ORF76 and ORF321, *tRNA^Trp^* sequence was 100% homologous to mitochondrial and chloroplast sequences. For ORF329, it was a one point mutation (Figure 4). Amino acid sequence alignment showed that there is a difference between amino acid sequences of ORF76 and ORF321 compared to ORF329 which could be a result of alternative splicing (Figure 4B).

*tRNA^Trp^* gene copies, as well as other four tRNA genes common for chloroplast and mitochondrial genomes, were located in chromosomes 1 and 4. For *tRNA^Trp^* it was also found in chromosomes 3 and 8, for *tRNA^Asp^* – in chromosome 5, for *tRNA^His^* – in chromosome 7, for *tRNA^Met^* – in chromosomes 3, 5, and 7, for *tRNA^Asn^* – in chromosome 7.

It was reported earlier that tRNAs in addition to their traditionally known role in translation might be involved in the regulation of transcript profiles (Irmer et al., 2010; Rogers et al., 2012). Computation analysis of tRNA sequences found in *V. faba* mitochondrion revealed that in the *M. truncatula* nuclear genome these tRNA sequences can be found as a part of different ORFs. Some of them were a part of exon or complementary to the part of exon; others were on the exon-intron junction point or a part of introns. All these sequences were part of transcripts, which suggests some active role. This role may be different in each specific case, but what attracted our attention is the fact that many of these tRNA sequence copies (not all) in *M. truncatula* nuclear genome were a part of some kind of transcribed ORF.

### Possible sequence relationship between plant mitochondrial and nuclear genomes

Summarizing data related to mitochondrial rrn5, rrn18, rps10, ATP synthase subunit alpha, cox2, and tRNA sequences as a part of nuclear transcribed ORFs led to the following conclusions:

1. *V. faba* mtDNA sequences can be organized into *Medicago truncatula* nuclear ORFs comprising various mitochondrial gene fragments. We present in this article genes *rps10*, *atpA* and *cox2*, ORF135, ORF134, and ORF198. But we found more such examples.
2. These ORF sequences are transcribed and spliced in the nuclear genome.
3. These ORFs may occur in nuclear genome in several versions representing variants of the same gene as result of alternative splicing or of recombination between ancestral ORFs.
4. Nuclear ORF genes comprise not only fragments of sequences encoding *V. faba* mitochondrial proteins but also *rrn5*, *rrn18*, or tRNA genes being analyzed in this work.
5. It is not clear whether rRNA or tRNA related sequences are translated (not previously reported) but their transcripts suggest a role in gene regulation.
6. We suggest that at least part of these ORFs could via gene duplication, recombination, and alternative splicing contribute to evolutionary innovation of genomes (Chen et al., 2012).

## Conflict of Interest Statement

The authors declare that the research was conducted in the absence of any commercial or financial relationships that could be construed as a potential conflict of interest.
